# Properly Classed

**Published:** 1895-10

**Authors:** 


### Properly Classed.

"Ya'as, I don't deny that I am an Anglomaniac. I thought you knew that, Miss Maud."

"I knew you were something of a maniac, Mr. Sissy, but didn't know what kind."—*Boston Beacon.*

